# Do our children learn enough in Sky Class? A case study: online learning in Chinese primary schools in the COVID era March to May 2020

**DOI:** 10.1186/s40561-021-00180-9

**Published:** 2021-12-16

**Authors:** Lina Zhao, Peter Thomas, Lingling Zhang

**Affiliations:** 1 Victoria University, Ballarat Rd Footscray, Melbourne, VIC 3029 Australia; 2 Communication University of Zhejiang, Hangzhou, China

**Keywords:** Sky Class, Physical class, Online learning, Primary school, Online technologies, Covid-19

## Abstract

All human being’s ways of living, working and studying were significantly impacted by the Covid-19 in 2020. In China, the Ministry of Education reacted fast in ensuring that primary school students could learn online at home by promoting the Sky Class program from February 2020. Educators, parents, and students all faced the challenges of adapting to new online teaching and learning environments. In this small-scale case study, Sky Class’s content and the participants’ experiences, will be presented. Four primary school teachers and five primary school students and their parents participated in three-rounds of interviews sharing their perspectives and experiences of online learning. The study showed that the students gained more parental support and that they benefited from using multimedia functions, like replay, in their Sky Classes. However, the majority of participants reported that the students learnt less. By mapping the learning activities and themes from Sky Class against Cope and Kalantzis’ e-learning ecologies, our study found that only ubiquitous learning and multimodal meaning were achieved. We suggest the reason may be that high cognitive learning was not achieved due to less teachers’ supervision, lack of interaction, delayed feedback, shorter learning times and communication. In conclusion, innovative pedagogies, which can foster different types of learning from the e-learning ecologies may overcome the negative aspects reported about Sky Class. Further research is required for implementing online technology as a catalyst for educational change.

## Introduction

The Chinese government reacted fast both in terms of controlling the pandemic and in continuing fundamental education nationally by promoting Sky Class programs. At 2 am on 23 January 2020, the central government of China issued a notice to start the “Wuhan Lockdown” to stop the outbreak of the coronavirus disease (Lockdowns rise, 2020). From 10 am, schools, companies, and all public transport, including buses, railways, flights, and ferry services were suspended. Four days after the Wuhan lockdown, the Ministry of Education directed authorities to shut down all schools nationally to prevent the spread of Covid-19 (Ministry of Education of People’s Republic of China, 2020b). The Sky class program of K-12 was developed eighteen days after the Wuhan lockdown. Primary schools which were thought of as the last field in education to be intruded on by online or distance learning, now had to accept this new teaching and learning form to cope with home quarantine and social distance policies.

This small-scale designed descriptive case study illustrates what Sky Class looked like, and the participants reactions to the use of online technology for meaningful learning in primary school sectors. The themes that were developed from interview data will address the following questions:

- Main research question: Did Chinese primary school students gain adequate learning through Sky Class program?
- Sub-question 1: What does a sky class look like?
- Sub-question 2: What were teachers, students, and their parents’ perceptions towards participating in online learning at the primary school level? Challenges and opportunities?

## Children and online learning

Online learning is defined as the implementation of teaching and learning practices in an online environment. It is a form of distance education, as learning occurs through the internet synchronously or asynchronously where students can learn collaboratively with their teachers and peers or independently by themselves regardless of time and space (Singh & Thurman, 2019; Yilmaz, 2019). Online learning allows both learners and teachers, who cannot attend a school, to learn due to Covid-19 social restrictions, to access education and educational information from different locations.

Online learning has grown fast during the past decade in many countries due to its advantages, resulting in educational change that shifts learners from physical face-to-face classrooms to virtual classrooms in universities (Aldhafeeri & Khan, 2016). First of all, online learning provides a flexible learning environment for students regardless of their physical location and availability which increased participation rates (Kim, 2020; Yilmaz, 2019). Secondly, online education offered a lower cost option for both education organization and students compared to in-person classes (Khurana, 2016; Kim, 2020; Yilmaz, 2019). In addition, many young children have been initiated into using digital technologies in their home lives so that the transition to engage in online learning activities is enhanced (Yelland, 2006).

However, online learning has limitations as many programs failed to provide the environment that engages students via active communication and social interaction (O'Doherty et al., 2018). Moreover, negative issues such as social isolation, lack of interaction and participation, and delayed feedback were raised in evaluation of online learning programs (Khurana, 2016). There are also limitations in implementing online technologies with young children for learning purposes. The increasing time that children spend in front of the screen might negatively impact their cognitive and physical development (Cordes & Miller, 2004; House, 2012). It is argued that children need to physically interact with their environments, for instance, it is important for them to engage in hands-on activities and physical play (House, 2012), as thinking develops from experience with concrete materials (a developmental perspective). This is because concrete materials in natural settings allows young children to be actively interactive (Cordes & Miller, 2000; Elkind, 2007). Online learning may not provide sufficient opportunities for young children to interact with physical objects through hands-on activities and physical play.

Another limitation to be considered is that young children’s readiness for participation in online learning is limited by their lack of abilities to access and use online learning systems effectively (Wedenoja, 2020). Moreover, adult supervision is required when young children participate in online learning and other types of online activities, therefore, adult availability and involvement also impacts young children’s online learning experiences (Kim, 2020; Youn et al., 2012). Nevertheless, with the unexpected crisis of Covid-19, the primary school teachers and students had to adopt online learning where “school and home’s spaces and times are mixed up” (Malta Campos & Vieira, 2021, p. 137).

Although, online learning or distance learning have been seen as viable alternative practices in the Covid-19 period, there is a lack of research exploring the implementation of online technologies in the primary school settings (Ching-Ting et al., 2014; Dong et al., 2020; Kerckaert et al., 2015). This study will help fill this gap by illustrating a full picture of online learning practices with young learners in China and examining Chinese primary students’ online learning experiences and their teachers, and parents’ perspectives towards online learning during the Covid-19 pandemic in 2020.

## Case study

This program utilised the case study method to collect and analyse the data of implementing online technology to support young students’ home learning during the Covid-19 quarantine in China. A case study suited the need to explore and examine “a contemporary phenomenon within its real-life, especially when the boundaries between a phenomenon and context are not clear and the researcher has little control over the phenomenon and context” (Yin, 2013, p. 13), by providing deep understanding, rich details and insights into participants’ experiences and perspectives (Billington, 2016).

### Participants

In this study, we invited participants who were currently employed as primary school teachers (teaching any subjects), and currently enrolled as primary school students (aged 6–13) and their parents who participated in Sky Class program for three-round interviews. A recruitment advertisement was posted on the WeChat social media circle, 46 adults participants showed their interests in participating the study. After a pilot interview of 46 teachers and parents, four teachers (see Table 1) who participated in recording sky class videos and live streaming class; and five families (see Table 2) whose children participated in the whole sky class program from the primary school sector were selected for interviews. The selected participants were considered to be the representative of the Sky Class program. One family from Shanghai only agreed to provide the Sky Class timetable from their children’s school. The rest of the participants were from Wuhan, Hohhot, Qingdao, Xi’an, and Zhejiang (see Tables 1, 2).

**Table 1:** Participant teachers’ information

Primary school teachers	Teaching subject	Region
Teacher Xue	English	Xi’an
Teacher Xuan	Literacy	Hohhot
Teacher Wang	Math	Wuhan
Teacher Liao	General	Wuhan

**Table 2:** The information of participant students and their parents

Primary school student	Parent	Year level	Region
Student Di	Parent Jia	Year Three (age 8)	Hohhot
Student Meng	Parent Wan	Year Two (age 7)	Wuhan
Student Tao	Parent Zhu	Year Five (age 11)	Qingdao
Student Mian	Parent Mao	Year Six (age 12)	Zhejiang
Student Yao	Parent Wang	Year Six (age 12)	Hohhot
Shanghai family		Year One	Shanghai
		The child and parents did not participate in the interview, but provided printed information of the Sky Class timetable	

### Data collection

Adults were initially interviewed for 30–50 min., and primary school students for 10–20 min. using sets of semi-open questions. Then, in the second and third round interviews (10–30 min.), they answered researcher’s follow up questions. All the interviews were conducted in Mandarin through video chat using the WeChat application and were recorded using the iPhone’s Voice Memos with password protection. Other digitalised data included online learning schedules, screenshots of applications and digital copies of online learning materials collected from the participants as well.

### Ethics

Parents’ consents were needed in this case study as the participant children were aged under 16. Moreover, one guardian was required to accompany their child in the interview to ensure that young children’s identities and rights were protected. All the participants’ identities were protected using anonymities. Any personal identifying information was not recorded, and accidental mentions were delated. Any data or information that participants wanted deleted were removed.

## Data analysis

The interviewed teachers, students and parents shared their perspectives, experience and stories of participating in Sky Class. Their interview data was put through an open coding method in the first-round analysis to capture the descriptive segments to address the sub-research question of what a sky class looked like. Then, during the second-round coding, the useful segments were further categorised into sub-themes as “lacking self-discipline”, “lacking interactions”, “lacking immediately feedback”, “short learning time”, “parents support” and “replay function” to address the sub-question of what the participants’ experience of and perceptions toward primary school students’ participation in online learning. These analyses indicated that in general, the participants had feelings of dissatisfaction towards the Sky Class; and concern that the primary school students might not engage in learning online effectively. They further concluded that the Sky Class could not replace physical classes at the primary school level. However, Sky Class has opportunities to be utilised as supplementary activities for physical classes enhancing young learner’s learning experiences after the pandemic.

### e-Learning ecologies and 7-affordences

The e-learning ecologies model and its e-affordances were utilised to assess the effectiveness of implementing online technology in Sky Class to see if new learning was promoted (Cope & Kalantzis, 2017). E-learning ecologies illustrates the possible innovative pedagogy patterns that can be designed for promoting new learning with digital technologies. Seven affordances were developed to demonstrate what new learning looks like with innovative and effective implementation pedagogies.

**Fig. 1:**
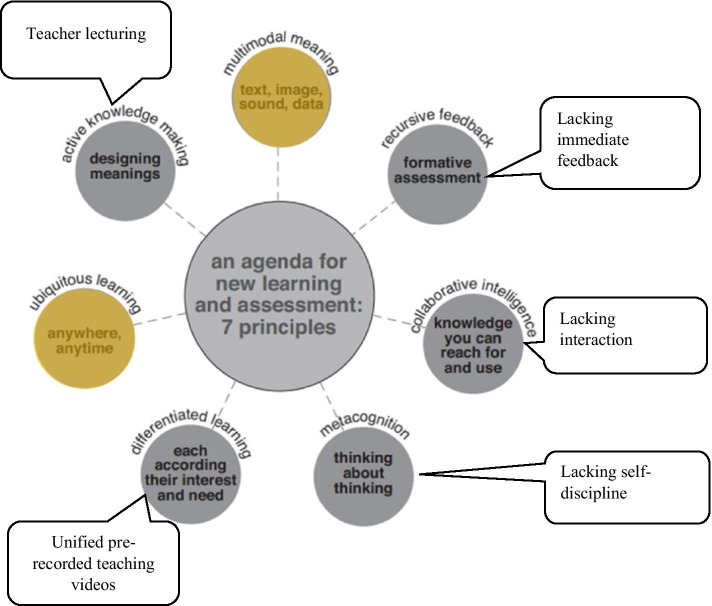
Mapping Sky Class learning activities and themes in e-learning ecologies (Cope & Kalantzis, 2017, p 14)

The annotated chart (see Fig. 1) was developed after examining the described learning activities and the resultant themes in the Sky Class to illustrate which affordances were addressed in the e-learning ecologies. We could only identify two affordances—ubiquitous learning and multimodal meaning—as the students were provided a wide range of digital technologies for accessing various multimedia resources such as teaching videos and live streaming classes (yellow-coloured circles in the Fig. 1). The rest of the affordances were hardly evidenced in our data. This showed that the innovative pedagogies that promoted independent, collaborative, critical, creative learning were missing in Sky Class online learning practices.

## Sky class

On 12th February 2020, the Ministry of Education officially issued a notice promoting a policy of “suspending classes without stopping learning” which encouraged all schools to provide the online learning programs called Sky Class to their students to learn from home (Ministry of Education of the People’s Republic of China, 2020a;  Zhang et al., 2020, XinHua News, 2020).

**Fig. 2:**
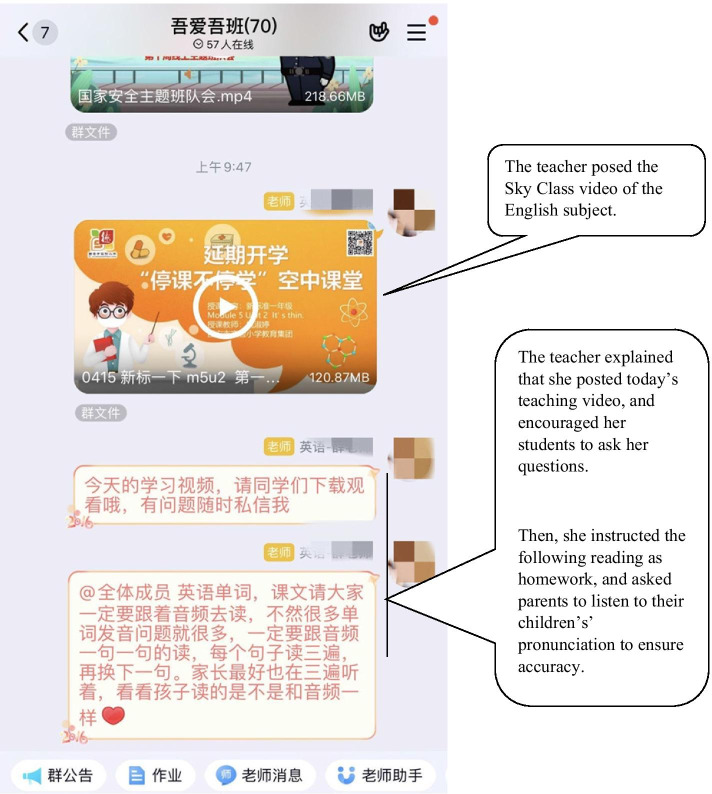
A screen shot of a participant teacher posting a Sky Class video and homework in her class QQ group chat

Based on our interview data, the Sky Class programs from the six regions are similar and mainly include asynchronous pre-recorded video teaching and synchronous live student Q&A torturing. The regional education departments gathered groups of teachers to develop sets teaching videos in literacy, numeracy and English for asynchronous online learning practices for their regional primary schools. The students can watch these pre-recorded teaching videos via TV and school websites daily based on their own schedules. Their classroom teachers also posted these videos files in their virtual classes that were established on WeChat or QQ program (see Fig. 2). Synchronous online leaning practices were promoted by each school from the six regions using student Q&A sessions which were live streaming teaching practices using Reng Reng Tong or the Teng Xun Class applications as a part of their regional Sky Class a few months after the Wuhan lockdown. All these practises ensured that students were able to access to sky classes by different devices based on their availabilities which indicated that ubiquitous learning from e-learning ecologies was successfully addressed in the Sky Class program.

However, in Hohhot region, the students from Year 3 and upper were required to attend the Sky Class. The students from Year 1–2 were still on holiday, and their major learning activities were finishing their holiday homework which was assigned by their classroom teachers in the last term of 2019.

### Content

The main form of the teaching videos for Sky Class are similar from the six regions according to our interview data, as they were PowerPoint slide shows (PPT) with teachers’ voices. More engaging teaching videos were produced a month later (from April 2020) by including teachers’ live images, cartons and 3D features, as the teachers received technique support and advice on recording and editing the videos. The camera function from the students’ side was not enabled in these sky classes at all. The students could participate in live streaming sky classes in some degree via microphone or chat box, to ask or answer questions synchronously. However, most of time they were passively involved in sky classes, as they just watched the screen silently. The teachers were still focused on orally explaining the teaching content in either recorded videos or live streaming classes which showed that the Chinese pedagogy of “teacher-centred lecturing” heavily impacted teachers’ practices on the Sky Class, indicating that the elements of active knowledge making and differentiated learning were not strongly reflected.

### Homework

**Fig. 3:**
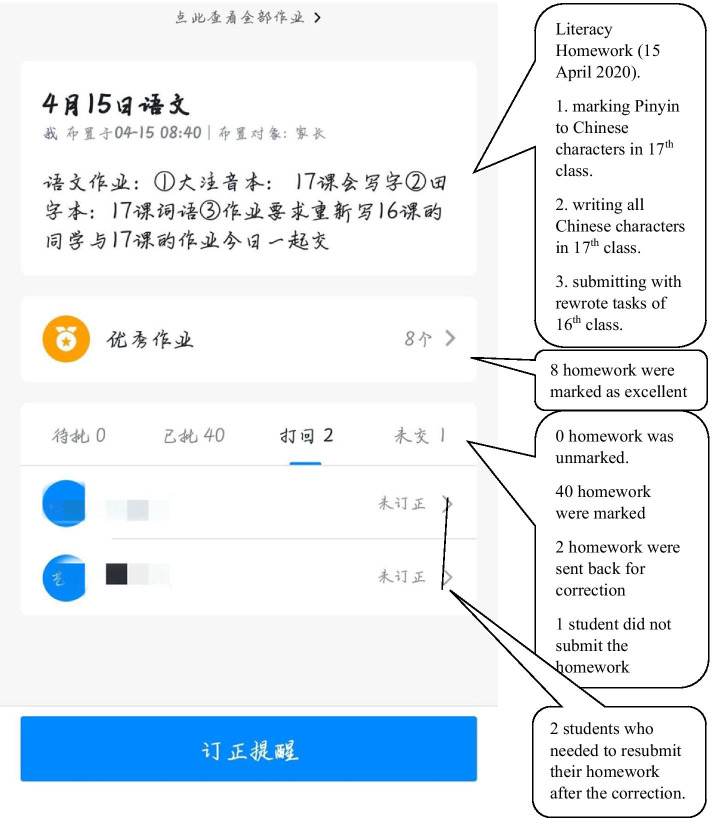
A screen shot of a participant teacher marking students’ homework digitally in Ding Ding application

The classroom teachers assigned the homework based on the learning intentions of each day’s Sky Class teaching videos on their virtual class (see Fig. 3). The students still used pen and paper to work on their tasks first. After they finished their work, they needed to photocopy their work and send them back to their teachers though virtual class portals with their parents’ assistance. The teacher marked these digital copies and provided the feedback on their virtual classes on smartphones or computers.

### Timetable

**Fig. 4:**
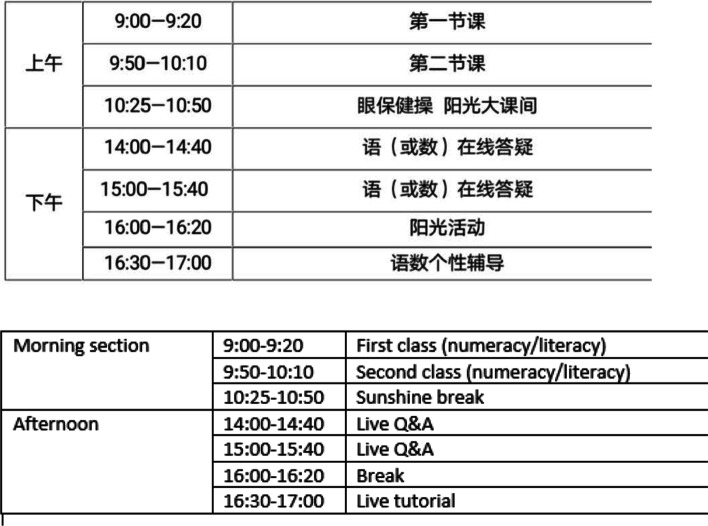
The sky class timetable in Wuhan region in April

**Fig. 5:**
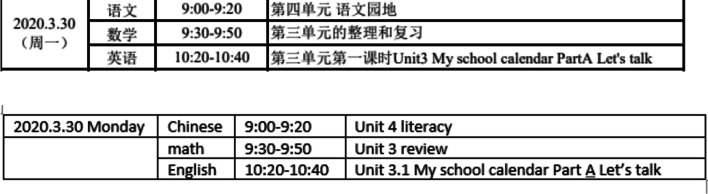
The sky class timetable in Hohhot region in March

By examining the timetables of the Sky Classes from the six regions, literacy, numeracy and English were the main focus and delivered in the morning session which was similar to the physical school’s timetables (see Figs. 4, 5). However, the total learning hours was decreased in Sky Class timetables compared to the physical school class timetable.

Each teaching video was only 20–30 min. long (45 min. each physical class) based on guidelines from the Ministry of Education that the teaching videos should not be too long for primary school students in terms of protecting their eye development and reducing the risk of short sightedness (Ministry of Education of People’s Republic of China, 2020a). Longer breaks (30–60 min.) between each sky class and one two-hour lunch break for a day were required by the Ministry of Education (Ministry of Education of People’s Republic of China, 2020a, 2020c). These requirements on the schedule of sky classes reduced actual learning time from 4–4.7 hr. per weekday in physical schools to 0.67–2.75 hr. online.

The participants also reported that the learning hours were shorter than the traditional school classes, and that they were concerned about shorter learning hours which might negatively impact young students learning. Student Yao said: “*Each class is short, so we might not learn that much*.” Teacher Xue reported that the poor quality of her students’ homework indicated that the students did not spend enough time on studying English. The participant parents were also concerned that their children could not absorb knowledge and information taught in the Sky Class due to such short learning times which might cause children to be left behind. Parent Zhu reported:

The learning time is obviously short. At school, they have 4 classes in the morning, and 3 classes in the afternoon. At home, 40 minutes in total for a sky class in a day. I do not think they can understand all the taught knowledge thoroughly in such a short time.

The evidence showed that teachers, students and their parents were concerned that less learning time was offered by the Sky Class, and that some students would be left behind as they learn less compared to what they had usually learnt in the physical class. Although there were many factors impacted the relationships between time and learning; Anderson (2018) reported that the students would perform lower degree of learning when they were not provided adequate learning time.

## Participants’ responses to the main research question

### The participants considered that young students failed to learn effectively due to poor self-discipline skills in the sky classes

Self-discipline was frequently emphasized by all the participants when they shared their experiences of participating in the Sky Class. Both teachers and parents believed that self-discipline was the key parameter to guarantee effective learning practise online which echoed Wang (2011) and Dörrenbächer and Perels (2016)’s statements that self-disciplined learning is a significant predictor for academic performance as learners can learn more effectively in an online learning environment when they developed sound skills in self-discipline and independence. The self-discipline in learning is defined as the process of moderating one’s thoughts, emotions and actions in a positive way to support learners to achieve their personal academic goals (Dörrenbächer & Perels, 2016). The participating Chinese primary school teachers provided their expectations of being “self-disciplined” for Sky Class with behavioural perspectives: being punctual, sitting still, concentrating, and finishing the homework. Less attentions were paid on pedagogies of improving students’ engagement.

However, primary school students reported that their abilities to regulate their learning online were limited which led to a situation of failing to develop a deep understanding of the Sky Class learning content. Teacher Wang reported that younger students found difficulty in concentrating on watching the videos independently due to a lower level of teachers’ supervision and control in the Sky Class, as good Sky Class learning always “*depends on their ability of self-discipline”.* However*,* most primary school students could not *“sit still for 20 min watching a teaching video.”* Teacher Xuan shared a same point of view that the major disadvantage of the Sky Class was that the students’ learning was not supervised online; therefore, “*a high level of self-discipline”* was required all the time to ensure students were learning using the online system. She also suggested that “*online learning might not the best learning mode for young students, especially primary school children”*.

The interviewed parents strongly agreed that their children were less pushed and disciplined for academical achievements in the Sky Class by their teachers than in physical schools. The parents complained that their children did not learn well in the Sky Class due to lacking self-discipline skills and believed that their children learn much better in physical classes as teachers could discipline their children to learn. Parent Wan said,

They need to be supervised and controlled all the time while learning. If you let them watch the sky class by themselves, they are just playing and not watching. My kids do not learn much if I do not supervise them. They have no self-discipline at home.

She further explained that her son who was in the Year One class attempted to play with iPads like “*fast forwarding*” the video rather than watching it for learning when he was not supervised.

Even the primary school students themselves reported that they found difficult in concentrating in the sky class. The Chinese primary school students reported that the teaching videos were not attractive, and they felt the lack of discipline from their teachers. “*Learning from home needs high level of self-discipline as it ensures you watch the whole video*. *Because teachers do not watch you during Sky Class.”* Student Ming said. Similarly, student Yao reported that she easily “*lost interests in watching Sky Class”.* Their reflections on the Sky Class showed that they had difficulty in disciplining themselves for learning online, and preferred to learn in physical classrooms. All these complains on lacking adult supervision were similar to what Kim’s study (2020) found which reported that young children’s online learning was heavily impacted by the adults who could provide supervision and support. Parents support in young children’s online learning during Covid period is essential (unicef.org, 2020). Young students need adults’ supervision and support to ensure high cognitive online learning practices.

### The participants considered that young students failed to learn effectively online due to limited interaction in the sky classes

As the Sky Class mainly included recorded teaching videos, many participants complained that there was almost no communication and interaction between students and teachers during the class. The interactions and communications among the classmates were even neglected, as the teachers claimed it would be very difficult to facilitate group tasks online with such age groups. Swan’s research shows it is important to create opportunities for interaction in online learning environments (Swan, 2010). This is because students learn more effectively when they can share their works and learning experiences with others which allow them to get feedback and more support from these people (Price, 2015).

The students reported that they felt bored and “*had no fun*” when they just watched recorded videos with zero communication and interaction with their teachers or other classmates. Although, in the physical classrooms, Chinese primary school students had limited communication and interaction with others, as they were required to be quiet and attentive during their teachers’ lecturing; they still had chances to put up the hands to answer questions, work with groups and model tasks for other students. The Sky Class reduced such limited communication and interaction to nearly none.

Student Di explained that the major aspect she did not like about Sky Class was zero communication and interaction with her Sky Class teachers. “*I always try to respond to my teacher’s questions in the video, but they cannot even hear me.*” She spoke. Meng’s mother complained that her children “*lost interests in watching teaching videos quickly due to zero interaction*.” It is suggested that interaction and communication in distance education were the critical predictors of student satisfaction which will lead to deep learning (Arbaugh, 2000).

Some of the participating teachers developed live streaming classes with more verbal immediacy behaviours to support interaction and communication among students and teachers. “*Using live streaming to teach is much better than using reordered teaching videos, as students can actually talk to their teachers and classmates*,” said Teacher Wang. However, teacher Liao further explained that “*although live streaming class is much better than recorded teaching videos*, *the level of communication in the live streaming class is still low compared to the communication level in the physical class.*”

The Chinese teachers were aware that interaction was reduced in sky classes, and they attempted to increase the level of communication and interaction by adopting live streaming technology. However, like Tao’s mother explained, the teacher could not have all the students on the microphone during their lecturing. Therefore, most of time, the students were watching the live streaming class silently.

We were also curious about how classmates communicated between each other in the Sky Class, and asked teachers about their opinions on student–student interactions in terms of supporting collaborative learning online. De-Verneil and Berge (2000) argued that encouraging discussion among students increased the student interaction and would lead to effective online learning, as “the learning process takes place within a social framework” (p. 236). However, all the interviewed teachers did not provide explanations on this question and continued to emphasise that student–teacher interaction and communication were important and needed to be addressed all the time. Break up groups or small group discussions were not promoted during the live streaming sky class which might enhance student–student communication and interaction. Peer support learning and facilitating peer support learning were neglected which showed that the full collaboration was not achieved in the Sky Class.

Other types of interaction such as the communication between teachers and teacher-parent were reported as significantly low in the Sky Class compared to physical schools. As “*it is not easy to hold video conference with our colleague teachers, but in school, we just pop into each other’s office to share our ideas and suggestions,*” teacher Xuan said. The parents also reported that they have less chance to communicate with their children’s teachers on the sky class as they only “*contact the teachers when it is a very important issue*”.

It is important to establish a collaborative learning environment which encourages communication, participation and interpretation between individuals and their communities (Rogoff, 2003). However, our study showed that the overall interaction and communication level in the Sky Class was very low which caused negative impacts on young students’ online learning experiences.

### The participants considered that young students failed to learn effectively online due to delayed feedback in the sky classes

The participant teachers reported that they were unable to give their usual on time feedback in Sky class to scaffold their students. They could only provide feedback on students’ homework which was obviously late compared with being in physical classrooms.

It is suggested that immediate feedback is one of the more effective assessment techniques that ensure students develop better understanding of online content (Gaytan & McEwen, 2007). Online assessment strategies include providing meaningful and timely feedback to students to guarantee powerful learning (Gaytan & McEwen, 2007). However, the participating teachers said that they did not teach their own classes online, as their students watched videos that were produced by others. The only way to know what and how well their students learnt was through marking their homework after the Sky Class. The teachers needed more days to identify their students’ learning needs, and the students got teachers’ feedback at least two days later. Teacher Xuan concluded that the Sky Class did not lead to effective learning compared to physical classroom, as teaching and learning did not happen “*on time*”.

Teacher Liao complained,

The assessment is always late, and you identify the problem two or three days’ later. Then you have to contact their parents. In school, you can immediately ask students to refresh the meaning of a word or grammar knowledge, but on the Sky Class or the live streaming class, you cannot.

Teacher Liao’s teaching experience showed that she considered providing on-time and on-site feedback was a significantly important strategy in engaging young learners in meaningful learning. Chinese teachers found difficulty in providing immediate feedback in sky classes which also occurred in adults’ online learning as a negative factor (Khurana, 2016). Obviously, effective feedback is not addressed well due to the low level of communication and interaction in the online classes which negatively impacts young learners learning experiences.

## Discussions

The themes, which were developed from analysing the interview data as well as relevant materials that were provided by the participants, indicated that both primary school teachers and students faced challenges in teaching and learning effectively in the online learning environment. Using the e-learning ecologies as a guide, only multimodal meaning and ubiquitous learning were realised in the sky class and more effort should be put on promoting other e-affordances for more effective online learning practices.

### The Chinese teachers are facing the challenges for developing alternative courses to accommodate their students in online environments

One possible reason was the novelty of the Sky Class or online environment to Chinese teachers. They were challenged to develop effective and appropriate teaching approaches, scaffolding strategies, teaching and learning resources, and tools to actively engage young students in the online learning environment in such short time. The teaching videos were developed simply and reinforced physical classroom’s teaching strategies such as “teacher lecturing” in sky classes which were less engaging when teachers were not physically present in front of their students. The classroom teachers only take the roles of passing on learning materials and marking the homework rather than effectively engage students in online learning practices using various digital tools and multimedia functions. However, according to Kim (2020), teachers and educators should develop appropriate and efficient strategies to ensure that their students are engaged in meaningful learning either online or offline. Even under the unexpected circumstances like quarantine restriction in Covid period, teachers are still required to react quickly enough to evaluate their lesson plans and modify these plans to develop the most appropriate and effective alternative courses for supporting their students learning (Kim, 2020).

Although the content of Sky Class is considered as important, the participant teachers reported that the adults’ support and scaffolding for learning were essential especially in the online learning environment. This is because learning occurs when students were scaffolded by more capable others when they attempt to accomplish more challenging tasks (Plowman & Stephen, 2013). However, these Chinese teachers over-emphasised on disciplining students’ behaviour in the Sky Class, including sitting still and concentrating on listening to the teaching videos, and finishing homework or tasks on time which were the same teaching strategies as in physical classrooms. The evidence echoes Tobin et al. (1989)’s results as they found that compared to other cultures, Chinese pedagogy emphasised more control and discipline. It seems that these teachers simply tried to replicate their physical classes online. None of the participant teachers expressed strategies or pedagogies to utilise advantages of the multimedia functions of digital technology to scaffold or train their students to learn effectively online. These teachers failed to address the affordances of metacognition and active meaning making as the learning activities in the Sky Class did not foster students’ skills for self-regulated learning, and limited their agency in learning.

### The Chinese teachers are facing the challenges to prepare their students ready for online learning

Another possible reason would be the Chinese pedagogy. The Chinese primary school students had already become used to learning via didactic and instructional approaches under teachers’ strict supervision in their physical classes. Few of them were taught the skills and strategies to or developed experiences of actively participating in self-directed online learning practices in their physical schools (Hsieh & Tsai, 2017). It is important for teachers to prepare their students with skills and knowledge in terms of accommodating challenges when they face unexpected circumstances such as suddenly changing learning environment in Covid period (Kim, 2017, 2020).

### The form of Sky Class did not promote activity learning

Also, the form of Sky Class limited its effective impact on learning as all the teaching videos were pre-recorded by other teachers reinforcing the teacher-lecturing pedagogy. Such form of online learning cuts off the effective communication between students and teachers and reduces synchronous online learning experiences which impacted young children’s learning negatively. Arnott and Yelland (2020) commented that the pedagogical approaches for implementing digital technologies are important for maximising the potential of digital technologies to empower young children's learning. However, our data showed that Sky Class failed to promote active knowledge making, and collaborative learning. Since digital technologies were mainly used for displaying learning content like voice embedded PPT videos and teacher lecturing live streaming presentations. Yelland (2015) further critiques that replicating existing pedagogical approaches to implement newest technology in the classroom does not release their potential and promote deep learning. In conclusion, the replication of Chinese pedagogy for physical classrooms in the Sky Class caused the insufficient implementation of digital technology which also led to ineffective learning.

### Possible improvements for Sky Class

Although there are many challenges Chinese primary school teachers have faced in the Sky Class program, the majority of participants reported that the program is necessary and essential for supporting primary school students to study at home during the Covid period. The students addressed that they liked the replay foundation of recorded teaching videos, as they could always go back to watch for better understanding of the learning concepts if needed.

There are few possible suggestions to improve the Sky Class program based on the themes that were developed from the interview data. As the students found difficult to maintain their attention while watching pre-recorded teaching videos, the strategy of increasing live streaming sessions each day may help students to sustain their attention for Sky class as they may gain more opportunities to communicate with their teachers through live-chatting. Enabling students’ cameras and microphones and developing small live chat groups will also allow them to communicate with their teachers and peers in a more effective way. In addition, the teachers can check their students’ learning processes and then offer the timely feedback in live streaming classes. Reducing the size of live streaming class will allow teachers to have more time and efforts on providing richer technology enriched learning environment for young students to interact with, as less effort and time will be spent on managing students’ online behaviours.

## Conclusion

The Sky Class was developed and promoted by the Ministry of Education associated with the quarantine policies for ensuring all students can participate in online learning at home safely in China.

Our study explored what online learning looked like by illustrating the Sky Class, and examined Chinese primary school teachers’, primary school students' and their parents’ perceptions, experiences and stories of participating in the Sky Class during the lockdown 2020. The results revealed that primary school students and teachers faced many challenges in organising online learning activities. The students appeared to lack the self-discipline skills necessary in this environment, and needed adults scaffolding in the Sky Class for meaningful learning. Rather than considering utilising digital technologies to scaffold and support young students’ learning, the Chinese primary school teachers put great emphasises on disciplining students’ behaviour in the online learning environment; and tried to replicate physical classrooms by implementing didactic teaching approach in the Sky Class. Didactic teaching approach and no face-to-face supervision contributed to an intensive focus on self-discipline. These reflected the Chinese pedagogy of control and regimentation and teacher-centred lecturing which is deeply rooted in early childhood and primary school education. It influenced teaching and learning behaviours even when the learning environment was switched to online. The possibilities of digital technologies in facilitating communication and interaction between student and teacher were not met, as pre-recorded teaching videos were the main teaching practices. There was little communication and interaction which led to delayed feedback and contributed to the participants’ dissatisfaction towards sky classes.

Although, young students were interested in the Sky Class and the live streaming classes, as such learning modes attracted their attention when digital technologies were used all the time; effective learning were not always guaranteed. It is necessary to design innovation online learning practices in early childhood and primary school settings, such as acknowledging the students' characteristics and learning needs for online learning, offering engaging learning materials, and particularly, providing effective scaffolding and feedback to optimize the online interaction (Kim & Neumann, 2020). A concentration on the innovative pedagogies promoted student-centred learning, collaboration, communication, and rich online learning environment to build e-learning ecologies would lead to more meaningful learning practices in the Sky Class.

## Data Availability

This study collected interviews as main data along with some phone screenshots and photos that were provided by the participants. All the data are highly confidential and will not release to any third parties unless the permissions are granted. Other materials including invitation letters, consent forms, and interview questions in Chinese are available from the corresponding author on reasonable request.

## Abbreviations

COVID-19: Severe acute respiratory syndrome coronavirus 2 (SARS-CoV-2)
QQ: A Chinese online chat program that is operated by Teng Xun company, also called as Teng Xun Chat in this paper
